# Arthroscopy-Assisted Reduction and Fixation of a Transversal Glenoid Fracture: About a Case

**DOI:** 10.1155/2017/2816216

**Published:** 2017-03-12

**Authors:** David Zbili, Eric Sali, Julien Serane, Edouard Lefèvre, Lior Amsallem

**Affiliations:** ^1^Service d'Orthopédie & Traumatologie, Hôpital Beaujon, 100 Boulevard du Géneral Leclerc, 92110 Clichy, France; ^2^Service d'Orthopédie & Traumatologie, Hôpital Saint-Antoine, 184 Rue du Faubourg Saint-Antoine, 75012 Paris, France

## Abstract

An articular glenoid fracture is an uncommon injury. Usually significantly displaced intra-articular glenoid fractures are treated with open reduction surgery. Conventional open surgery techniques involve high morbidity. Here we describe an arthroscopy-assisted reduction and fixation method of an Ideberg type III glenoid fracture. This method provides good articular reduction without extensive exposure or soft tissue dissection and without nerve and/or vascular lesion.

## 1. Introduction

**Figure 1:**
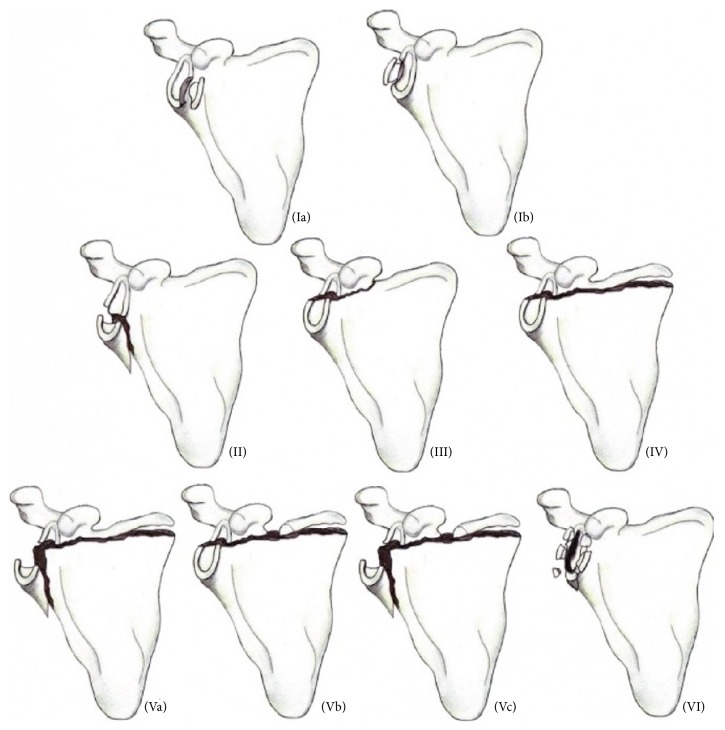
Ideberg classification.

Glenoid fractures are uncommon and represent 10% of scapula fractures [1]; only 1% involve significant displacement [2]. These fractures usually occur following high energy trauma and include bone and tissue lesions. Several classifications have been described to establish management protocols for this rare pathology. The most widely used is Ideberg [3]; see Figure 1.

Orthopedic treatment for displaced glenoid fractures results in poor functional results and short- or long-term evolution towards osteoarthritis. Thus, most of these fractures are treated by open reduction and internal fixation [4]. Displaced glenoid articular fracture surgery is difficult and invasive and involves a large approach resulting in muscular tissue damage [2, 5].

Recently, minimally invasive arthroscopic techniques have allowed for optimal visualization and reduction in joint surfaces and have given good results [6, 7].

The arthroscopic approach has been described mostly for anterior glenoid fractures, using suture anchor techniques for the control and fixation of the fragment, rarely by percutaneous screw fixation [8, 9].

Here, we describe the case of an Ideberg type III glenoid fracture treated by minimally invasive arthroscopic technique.

## 2. Case Report

**Figure 2:**
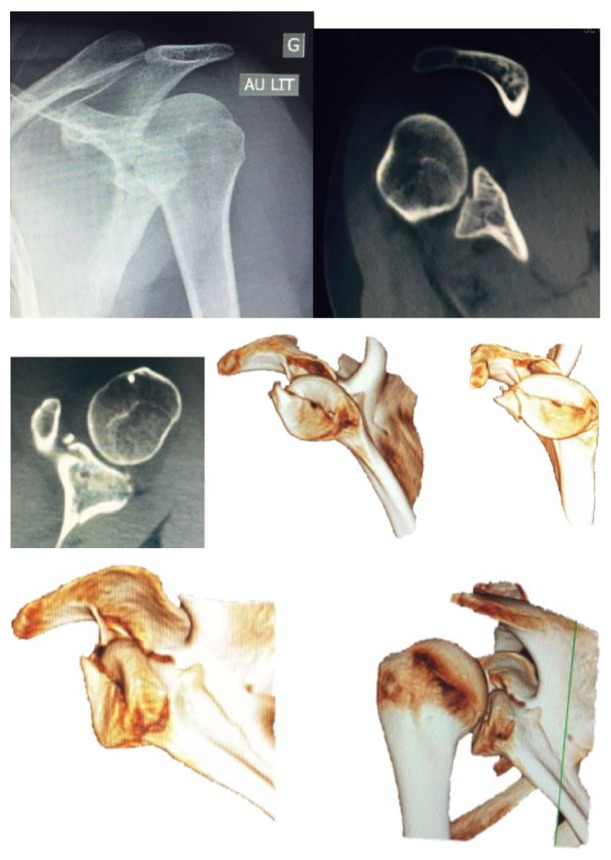
X-ray and CT with 3D images of an articular glenoid fracture.

A 22 y/o right-handed female student was involved in a high energy trauma motorcycle accident. Clinical exam results showed injury to the left shoulder involving painful total loss of function without vascular or nerve deficit of the arm and a left hemopneumothorax. X-ray and CT imaging with 3D reconstructions showed a transverse glenoid fracture that separated the upper one-third of the glenoid fossa, characterizing a type III Ideberg fracture. It also showed an articular surface irregularity of more than 5 mm (Figure 2).

The fracture was treated by arthroscopy with a canulated screw fixation and reduction using the Arthrex Tightrope ancillary three days after the accident.

## 3. Surgical Technique

Under general anesthesia, the patient was placed in a beach-chair position without traction of the left arm. An image intensifier was placed above the shoulder in a fashion allowing the X-ray beam to be centered on the glenoid. Bone structures and approaches (anterior, posterior, and Neviaser) were traced using a marker. After drapes were applied, we used a posterior approach to explore the glenohumeral cavity which allowed us to see the transverse fracture line, separating the superior third of the glenoid. Manipulation and reduction of the proximal fragment were facilitated by using a first percutaneous K-wire in the glenoid, which gave support for the probe and spatula which were inserted through an anterior approach through the rotator interval. Evacuation of the haematoma and clots using a shaver allowed for visualization of the reduction criterion. After reduction using the probe and the K-wire, visual and image intensifier control showed proper reduction of the fragment. The Arthrex Tightrope ancillary was inserted through the anterior and Neviaser approaches and placed above and under the glenoid in the reduction position, allowing insertion of a guide wire for the 4.5 mm canulated Biotech screw (Omnitech).

**Figure 3:**
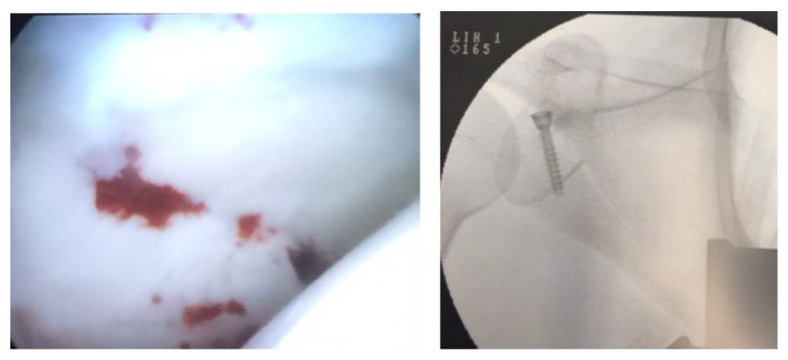
Arthroscopic and fluoroscopic view.

The canulated screw was placed after sequential drilling, allowing for compression and preventing rotation of the fragment. Proper and stable reduction was obtained and confirmed visually and through image intensifying (Figure 3).

Immediate postoperative management consisted in immobilization for 6 weeks without rehabilitation due to the comminute nature of the scapula. Following this period, passive and active rehabilitation was undertaken.

**Figure 4:**
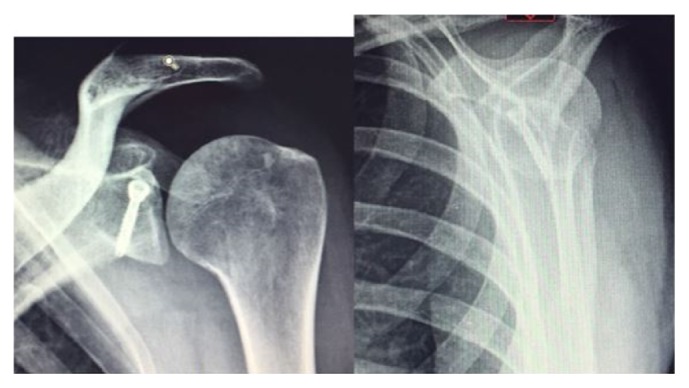
AP and lateral X-ray at 6-month follow-up.

Three month postoperative X-rays showed complete consolidation, and clinical examination revealed no axillary or suprascapular palsy (Figure 4).

At six months, muscle force and mobility were complete and symmetrical, with anterior elevation at 170°, 160° abduction, external rotation at 40°, and internal rotation at T10.

At last follow-up of one year, the UCLA shoulder score was of 35 out of 35, the Constant functional score was 100 out of 100, and a converted quick DASH score was 0 out of 100.

## 4. Discussion

Glenoid fractures, as all joint fractures, require anatomic reduction with stable fixation in order to avoid potential evolution towards osteoarthritis and chronic pain [10]. Apart from anteroinferior glenoid fractures, observed in 20 to 57% of all acute shoulder dislocations [11], glenoid fractures remain extremely uncommon [12]. Even if orthopaedic treatment seems to be less invasive, meta-analyses recommend reduction and internal fixation for displaced fractures of more than 5 mm, which was the case for our patient [4, 12, 13]. Usually, treatment for this type of fracture consists in open reduction allowing for visual control. Surgical approach is typically posterior and causes important muscle damage, risk of axillary and suprascapular nerve damage, and postoperative muscle weakness [4, 14].

Arthroscopic techniques are used widely for different joint fractures as for tibial plateau fractures [6] and wrist fractures [7]. As concerns the shoulder, these techniques have shown to be efficient in fractures of the greater tubercle and in fractures of the external quarter of the clavicule.

Several publications have shown the efficiency of arthroscopic treatment of glenoid fractures in general. Bauer et al. [8] described a transglenoidal anchor osteosuture technique after reduction by probe for type Ia and Ib Ideberg fractures. Tauber et al. [15] described the same technique by using a canulated screw for compression with excellent clinical results.

For type III Ideberg fractures, only two studies have described arthroscopic treatment with compression screw fixation, as for our case. Yang et al. reported on a series of 18 cases, with an 18-month follow-up, with an average Constant score of 96.8 at the last follow-up and a UCLA score of 34.3 [16]. Bonczek et al. describe a case similar to ours with excellent functional and radiological results after one year [17]. However, their method of reduction of the fracture was not similar to ours. In their different methods, they used the coracoid for support in reduction after a brief deltopectoral approach. Our patient had a coracoid fracture; thus reduction was done with a K-wire and arthroscopy probe.

The risk of neurovascular lesions during percutaneous glenoid synthesis has been well described by Marsland and Ahmed in their cadaveric study. It showed that the upper part of the glenoid is accessible for percutaneous screw fixation between quadrants 7h40 and 2h50 [18]. Percutaneous screw fixation using the upper Neviaser approach, as was done with our patient, is feasible and risk-free.

## 5. Conclusion

We have presented the case of a transversal articular glenoid fracture treated by arthroscopic percutaneous screw fixation. This method seems to give good functional and radiological results without risk, on the short to long term, for this type of fracture.

## References

[1] Cole P. A., Freeman G., Dubin J. R. (2013). Scapula fractures. *Current Reviews in Musculoskeletal Medicine*.

[2] Adam F. F. (2002). Surgical treatment of displaced fractures of the glenoid cavity. *International Orthopaedics*.

[3] Van Oostveen D. P. H., Temmerman O. P. P., Burger B. J., Van Noort A., Robinson M. (2014). Glenoid fractures: a review of pathology, classification, treatment and results. *Acta Orthopaedica Belgica*.

[4] Schandelmaier P., Blauth M., Schneider C., Krettek C. (2002). Fractures of the glenoid treated by operation. A 5- to 23-year follow-up of 22 cases. *Journal of Bone and Joint Surgery—Series B*.

[5] Qin H., Hu C.-Z., Zhang X.-L., Shen L.-X., Xue Z.-C., An Z.-Q. (2013). Surgical treatment of Ideberg type III glenoid fractures with associated superior shoulder suspensory complex injury. *Orthopedics*.

[6] Caspari R. B., Hutton P. M., Whipple T. L., Meyers J. F. (1985). The role of arthroscopy in the management of tibial plateau fractures. *Arthroscopy: The Journal of Arthroscopic and Related Surgery*.

[7] Abe Y. (2014). Plate presetting and arthroscopic reduction technique (PART) for treatment of distal radius fractures. *Handchirurgie, Mikrochirurgie, Plastische Chirurgie*.

[8] Bauer T., Abadie O., Hardy P. (2006). Arthroscopic treatment of glenoid fractures. *Arthroscopy: The Journal of Arthroscopic & Related Surgery*.

[9] Bauer T., Soubeyrand M., Hardy P. (2006). Traitement arthroscopique des fractures articulaires de l'épaule. *Chirurgie de la Main*.

[10] Anderson L. D. (1965). Compression plate fixation and the effect of different types of internal fixation on fracture healing. *The Journal of Bone & Joint Surgery*.

[11] Trillat A., Dejour H., Roullet J. (1965). Recurrent luxation of the shoulder and glenoid labrum lesions. *Revue de Chirurgie Orthopedique et Reparatrice de l'Appareil Moteur*.

[12] Goss T. P. (1992). Current concepts review. Fractures of the glenoid cavity. *Journal of Bone and Joint Surgery. Series A*.

[13] Lantry J. M., Roberts C. S., Giannoudis P. V. (2008). Operative treatment of scapular fractures: a systematic review. *Injury*.

[14] Anavian J., Gauger E. M., Schroder L. K., Wijdicks C. A., Cole P. A. (2012). Surgical and functional outcomes after operative management of complex and displaced intra-articular glenoid fractures. *The Journal of Bone & Joint Surgery—American Volume*.

[15] Tauber M., Moursy M., Eppel M., Koller H., Resch H. (2008). Arthroscopic screw fixation of large anterior glenoid fractures. *Knee Surgery, Sports Traumatology, Arthroscopy*.

[16] Yang H.-B., Wang D., He X.-J. (2011). Arthroscopic-assisted reduction and percutaneous cannulated screw fixation for Ideberg type III glenoid fractures: a minimum 2-year follow-up of 18 cases. *American Journal of Sports Medicine*.

[17] Bonczek S. J., Hutchinson R., Chakravarthy J. (2015). An innovative method of fracture reduction in an arthroscopically assisted cannulated screw fixation of an Ideberg type III glenoid fracture. *International Journal of Shoulder Surgery*.

[18] Marsland D., Ahmed H. A. (2011). Arthroscopically assisted fixation of glenoid fractures: a cadaver study to show potential applications of percutaneous screw insertion and anatomic risks. *Journal of Shoulder and Elbow Surgery*.

